# Differential Effects of Nitrostyrene Derivatives on Myelopoiesis Involve Regulation of C/EBPα and p38MAPK Activity

**DOI:** 10.1371/journal.pone.0090586

**Published:** 2014-03-10

**Authors:** Marije Bartels, Andrana K. Calgarotto, Anton C. Martens, Victor Maso, Saulo L. da Silva, Marc B. Bierings, Mary L. de Souza Queiroz, Paul J. Coffer

**Affiliations:** 1 Department of Cell Biology, University Medical Center Utrecht, Utrecht, The Netherlands; 2 Division of Pediatrics, University Medical Center Utrecht, Utrecht, The Netherlands; 3 Departamento de Farmacologica, Universidade Estadual de Campinas, Campinas/SP, Brazil; 4 Department of Immunology, University Medical Center Utrecht, Utrecht, The Netherlands; 5 Departamento de Química, Universidade Federal de São João Del-Rei, Ouro Branco/MG, Brazil; Faculté de médecine de Nantes, France

## Abstract

Bone marrow failure syndromes and MDS represent a heterogenous group of diseases, characterized by ineffective myelopoiesis, the risk of clonal evolution and a generally poor response to chemotherapy-based treatment regimen. Nitrostyrene derivatives have been studied as protein phosphatase inhibitors in various tumor models. Pharmacological studies have identified nitrostyrene as the structural core underlying a pro-apoptotic effect in tumor cells, yet their effects on normal cells, including those of the hematopoietic system, are largely unknown. In this study, utilizing umbilical cord blood-derived myeloid progenitor cells, patient-derived bone marrow cells, and a (BALB/c) mouse model; we investigated the effects of treatment with two nitrostyrene derivatives (NTS1 and NTS2) on myeloid development. We demonstrate that these compounds stimulate the expansion and differentiation of myeloid progenitors *in vitro* and improve myeloid reconstitution after chemotherapy-induced bone marrow depletion *in vitro* and *in vivo*. These effects were accompanied by increased C/EBPα expression and activity and inhibition of the p38MAPK signalling pathway. Together, our data suggest that nitrostyrenes improve myelopoiesis and represent potential new treatment strategies for patients suffering from bone marrow failure syndromes, hypocellular myelodysplastic syndrome and chemotherapy-induced aplasia.

## Introduction

Hematopoiesis is a carefully orchestrated process involving self-renewal and differentiation of primitive pluripotent stem cells and resulting in the formation of blood cells [1]–[4]. Differentiation of common myeloid progenitors (CMP) generates cells of both the granulocyte/macrophage lineage, leading to the formation of granulocytes, monocytes and macrophages, as well as the megakaryocyte/erythroid lineage, leading to the formation of megakaryocytes, platelets and erythrocytes. Neutrophil and monocyte/macrophage development is tightly regulated by key transcription factors including C/EBPα and PU.1 [5]–[7] and in recent years it has become clear that the expression and function of such proteins is regulated by post-translational modifications, including phosphorylation by members of the c-Jun N-terminal kinases (JNK), extracellular signal-regulated kinases (ERK) and p38-mitogen activated protein kinases (MAPK) pathways, which has resulted in increased understanding of the regulation of normal and aberrant myelopoiesis over the past decade [8],[9].

In the search for new cancer therapies, the effects of nitrostyrene derivatives have been investigated in various human and non-human tumor models. Mechanistic studies of these compounds in tumor cells have demonstrated that nitrostyrenes have pro-apoptotic effects based serine/threonine phosphatase (PP2A) inhibition [10],[11], while other studies have proposed that nitrostyrenes can function as telomerase inhibitors [12], phospholipase (A2) inhibitors [13], tyrosine phosphatase inhibitors (PTP1B, SHP1, Yop) [14], or tyrosine kinase inhibitors (Src, Syk, FAK) [15]. Recently, we have studied the effects of the nitrostyrene derivatives NTS1 and NTS2 on tumor growth and survival in the Ehrlich ascites tumor (EAT) model [16] *in vivo* (unpublished data). Here, in addition to the effects of NTS1 and NTS2 on tumor survival, we observed an increase in the formation of myeloid colony forming units (CFU) from isolated bone marrow (BM) mononuclear cells, suggesting that NTS1 and NTS2 stimulate myeloid regeneration following bone marrow suppression. Based on these results, the mechanistic studies with nitrostyrenes and the knowledge concerning the functional role of MAPK signalling pathways, we hypothesized that the effects of NTS1 and NTS2 on myelopoiesis could involve modulation of serine/threonine phosphatase, or kinase (MAPK) activity and their substrates.

In this study, utilizing an *ex* v*ivo* myeloid differentiation system as well as a mouse model, we demonstrate that treatment with NTS1 and NTS2 induces a dramatic increase in myeloid progenitor expansion and differentially regulates granulocyte/macrophage lineage development *in vitro* and *in vivo*. These effects were accompanied by increased C/EBPα expression and reduced C/EBPα phosphorylation, which can at least in part be explained by inhibition of p38MAPK activity. Together our data illustrate a potential role for nitrostyrene derivatives in the development of new treatment strategies in myeloid disorders, including bone marrow failure (BMF), myelodysplastic syndrome (MDS) or chemotherapy-induced aplasia.

## Materials and Methods

### Nitrostyrene compounds

The nitrostyrenes 1-((E)-2-nitrovinyl) benzene (NTS1) and 1-nitro-3-((E)-2-nitrovinyl) benzene (NTS2) were synthesized by Villar *et al*. by procedures described in literature [13] and kindly donated. NTS1 and NTS2 were selected out of ten nitrostyrene derivatives based on the results in the EAT model (unpublished data), stored in dimethyl sulfoxide (DMSO) and diluted 1000x in Isocove's Modified Dulbecco's Medium (IMDM) before usage.

### Mice

Male BALB/c mice, 6–8 weeks old, were bred and maintained under specific pathogen-free (SPF) conditions at the University Central Animal Facilities (Centro de Bioterismo, Universidada Estadual de Campinas, Campinas, SP, Brasil). Mice were maintained in cages (4 mice per cage) on shavings in a conditioned room with a light/dark cycle of 12 hours, a temperature of 25°C, standard chow (Nuvilab) and filtered water freely available. Anaesthesia was performed utilizing Xylazine (10 mg/kg) and Ketamine (80 mg/kg) (Sigma-Aldrich, Seelze, Germany) and mice were euthanized through deep anaesthesia. Animal experiments were performed in accordance with institutional protocols and guidelines of the Institutional Animal Care and Use Committee, in agreement with the recommendations of the Canadian Council on Animal Care. Approval was obtained from the Committee on the Ethics of Animal Experiments of the State University of Campinas. All efforts were made to minimize suffering.

### Patients

Bone marrow specimens were collected during a yearly control visit and bone marrow examination (patient 1) or during control bone marrow examination four weeks after chemotherapy treatment for AML (patient 2 and 3). Written informed consent was obtained from patient 1 and all parents (patient 1–3) according to the Declaration of Helsinki. Protocols were approved by the ethics committee of the University Medical Center Utrecht.

### Isolation and culture of human CD34+ cells

Mononuclear cells (MNC) were isolated from human umbilical cord blood by density centrifugation over a Ficoll-Paque solution (density 1.077 g/mL). MACS immunomagnetic cell separation (Miltenyi Biotech, Auburn, CA, USA) using a hapten-conjugated antibody against CD34, which was coupled to beads, was used to isolate CD34+ cells. 5.0×10^4^ CD34+ cells were cultured in Iscove's modified Dulbecco's medium (Gibco, Paisley, United Kingdom) supplemented with 8% fetal calf serum (FCS) (Hyclone, South Logan, Utah, USA), 50 μmol/L of β-mercaptoethanol, 10 units/mL of penicillin, 10 µg/mL of streptomycin, and 2 mM glutamine at a density of 0.3×10^6^ cells/mL. Cells were differentiated towards neutrophils in 17 days upon addition of stem cell factor (SCF) (50 ng/mL), FLT-3 ligand (50 ng/mL), granulocyte macrophage colony-stimulating factor (GM-CSF) (0.1 nmol/L), interleukin 3 (IL-3) (0.1 nmol/L), and granulocyte colony-stimulating factor (G-CSF) (30 ng/mL). Every 3 days, cells were counted with trypan blue, and fresh medium was added to a density of 5.0×10^5^ cells/mL. After 3 days of differentiation only G-CSF was added to the cells. NTS1 and NTS2 (0.5–5 µM) were added to the fresh medium every 3 days. The NTS concentrations were selected based on the results in the EAT model (unpublished data). Umbilical cord blood and bone marrow was collected after written informed consent was provided according to the Declaration of Helsinki. Protocols were approved by the ethics committee of the University Medical Center Utrecht.

### Flowcytometric analysis

Cells were isolated after 3, 7 and 10 days of neutrophil differentiation and washed with PBS. Samples were subsequently incubated for 15 minutes with AnnexinV-FITC (Bender MedSystems, Vienna, Austria) in binding buffer (10 mmol/L HEPES-NaOH (pH 7.4), 150 mmol/L NaCl, 2.5 mmol/L CaCl_2_). Cells were washed and resuspended in binding buffer containing 1 μg/mL propidium iodide (Bender MedSystems, Vienna, Austria). Percentages of early apoptotic (Annexin V-positive, propidium iodide- negative) and late apoptotic (Annexin V- and propidium iodide-positive) cells were determined by FACS analysis (FACS Canto, Becton Dickinson, Alphen a/d Rijn, The Netherlands).

To analyze the percentage of CD34+ cells, cells were isolated after 3, 7 and 10 days of differentiation, subequently washed and resuspended in PBS/5% FCS (Hyclone, South Logan, Utah, USA). Next, cells were incubated for 30 min on ice with a phycoerythrin conjugated CD34 antibody (Becton Dickinson, Alphen a/d Rijn, the Netherlands). After incubation, cells were again washed and the percentage of CD34-positive cells was determined by FACS analysis.

Lactoferrin staining was used to analyze neutrophil differentiation after 17 days. Cells were isolated, fixed in 100 µL 0.5% formaldehyde for 15 minutes at 37°C, followed by permeabilization in 900 µL of ice-cold methanol for 30 minutes on ice. Cells were subsequently washed with PBS, and incubated with phycoerythrin (PE)-conjugated lactoferrin antibody (Immunotech, Marseille, France) for 25 minutes. Cells were again washed and FACS analysis was performed.

### CFU assay with CD34+ cells from UCB or BM

CD34+ cells after isolation (500 cells per condition) and after three days of differentiation (1000 cells per condition) were isolated and plated in IMDM (Gibco, Paisley, United Kingdom) supplemented with 35.3% FCS (Hyclone, South Logan, Utah, USA), 44.4% methylcellulose-based medium (Methocult, StemCell Technologies, Vancouver, Canada), 11.1 μmol/L of β-mercaptoethanol, 2.2 units/mL of penicillin, 2.2 μg/mL of streptomycin, and 0.44 mmol/L of glutamine. CFU assays were performed in the presence of SCF (50 ng/mL), FLT-3 ligand (50 ng/mL), GM-CSF (0.1 nmol/L), IL-3 (0.1 nmol/L), G-CSF (60 ng/mL) and EPO (6IE/mL). NTS1 and NTS2 were added to the medium in a single dose. CFU-GM, CFU-G, CFU-M and CFU-E colonies were scored after 11 days of culture.

### Cytochemical staining of myeloid cells

May-Grunwald Giemsa staining was used to analyze myeloid differentiation. Cytospins were prepared from 5.0×10^4^ differentiating granulocytes and were fixed in methanol for 3 minutes. After fixation, cytospins were stained in a 50% eosin methylene blue solution according to May-Grunwald (Sigma Aldrich, Seelze, Germany) for 15 minutes, rinsed in water for 5 seconds, and nuclei were counterstained with 10% Giemsa solution (Merck kGaA, Darmstadt, Germany) for 20 minutes. Neutrophil differentiation can be characterized by distinct stages from myeloblast, promyelocyte I, promyelocyte II, myelocyte and metamyelocyte towards neutrophils with banded or segmented nuclei. Mature neutrophils were characterized as cells containing either banded or segmented nuclei. Micrographs were acquired, after staining with May-Grunwald Giemsa solution, with an Axiostar plus microscope (Carl Zeiss, Sliedrecht, the Netherlands) fitted with a 100x/1.3 NA EC Plan Neofluor oil objective using Immersol 518F oil (Carl Zeiss), a Canon Powershot G5 camera (Canon Nederland, Hoofddorp, the Netherlands), and Canon Zoombrowser EX image acquisition software. Photoshop CS3 was used for image processing (Adobe Systems Benelux, Amsterdam, The Netherlands).

### Western blot analysis

Western blot analysis was performed using standard techniques utilizing antibodies directed against phosphorylated ERK1/2 (Thr202/Tyr204), ERK1/2, phosphorylated p38, p38, phosphorylated C/EBPα (Ser21) (all from Cell Signalling Technology, Beverly, MA, USA), or C/EBPα (Santa Cruz Biotechnology, Santa Cruz, CA, USA). An antibody directed against tubulin (Sigma-Aldrich, Seelze, Germany) was used as a loading control.

### Analysis of myelopoiesis in BALB/c mice following 5-fluorouracil (5FU)–induced bone marrow depletion

BALB/c mice were treated intraperitoneally with 150 mg/kg 5-FU (Sigma, St.Louis, MO, USA) at day 0 according to Rich [17]. Mice were treated intraperitoneally with 1 mg/kg NTS1 or NTS2 once a week until day 21 after 5-FU treatment. Mice treated with 5FU only were used as controls. After 2, 5, 9, 15 and 21 days, mice were sacrificed, bone marrow MNC were isolated by flushing both femurs, followed by CFU assays with 5×10^4^ MNC per condition. CFU-G and CFU-M were scored after 7 days.

### Statistics

Statistical analysis was performed using a one-way ANOVA test followed by a Dunnet multiple comparison test to compare the differences between the control and NTS-treated cells in all experiments (Prism GraphPad Software). *P*-values of 0.05 or less were considered significant (*p = <0.05, **p = <0.01*).

## Results

### NTS1 and NTS2 have concentration dependent effects on neutrophil progenitor expansion and survival

**Figure 1:**
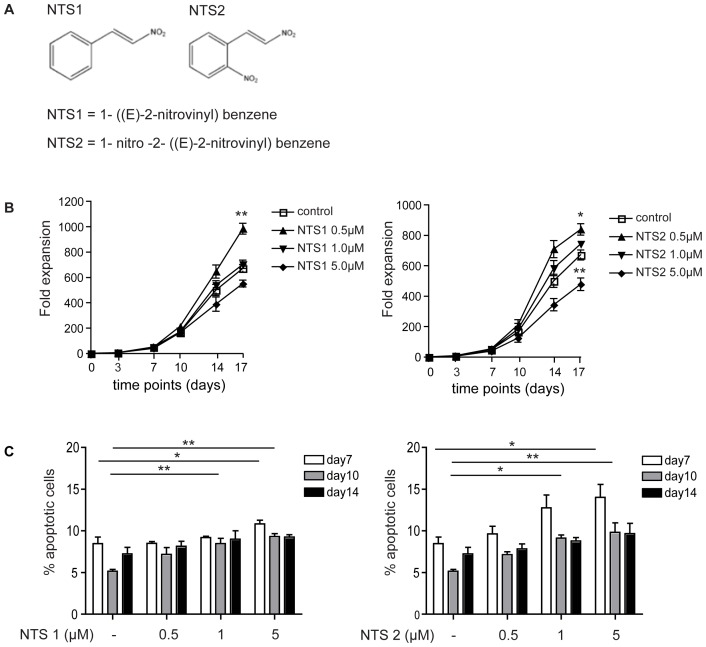
NTS1 and NTS2 have concentration dependent effects on neutrophil progenitor expansion and survival. CD34+ cells were cultured in presence of G-CSF to induce neutrophil differentiation. Cells were cultured either in the absence or presence of NTS1 (0.5–5 µM) or NTS2 (0.5–5 µM) (**A**). Progenitor expansion was determined by counting the tryphan blue negative cell population. Data were expressed as fold expansion (**B**) (N = 4). Apoptosis was determined by Annexin-V/PI staining at day 7, 10, and 14 and data were expressed as the percentage of apoptotic cells (**C**) (N = 3). Error bars represent SEM (between experiments) *p = <0.05, **p = <0.01.

The nitrostyrene derivatives NTS1 and NTS2 were synthesized by procedures previously described (13) (Figure 1A). To investigate the effects of nitrostyrene derivatives on human myeloid development, we utilized an *ex vivo* differentiation system, in which UCB-derived CD34+ hematopoietic progenitor cells were differentiated towards neutrophils in the presence of G-CSF. To determine the effects of NTS treatment on neutrophil progenitor expansion and viability, we cultured cells in the absence or presence of NTS1 or NTS2 (0.5–5.0 µM). Treatment of neutrophil progenitors with 0.5 µM NTS1 and NTS2 resulted in a significant increase in progenitor expansion, while treatment with 5.0 µM NTS2 resulted in a significant decrease in progenitor expansion (Figure 1B). Compared to the control and treatment with 0.5 µM NTS1 or NTS2, the effects of higher concentrations of NTS compounds, in particular NTS2, were accompanied by a significant increase in the percentage of apoptotic cells at day 10 of differentiation (Figure 1C). Together, these data demonstrate that NTS1 and NTS2 have concentration dependent effects on neutrophil progenitor proliferation and survival of neutrophil precursors, and suggest that treatment with lower concentrations of NTS1 and NTS2 stimulates myeloproliferation.

### NTS1 and NTS2 differentially stimulate myeloid progenitor expansion and granulocyte/macrophage lineage development

**Figure 2:**
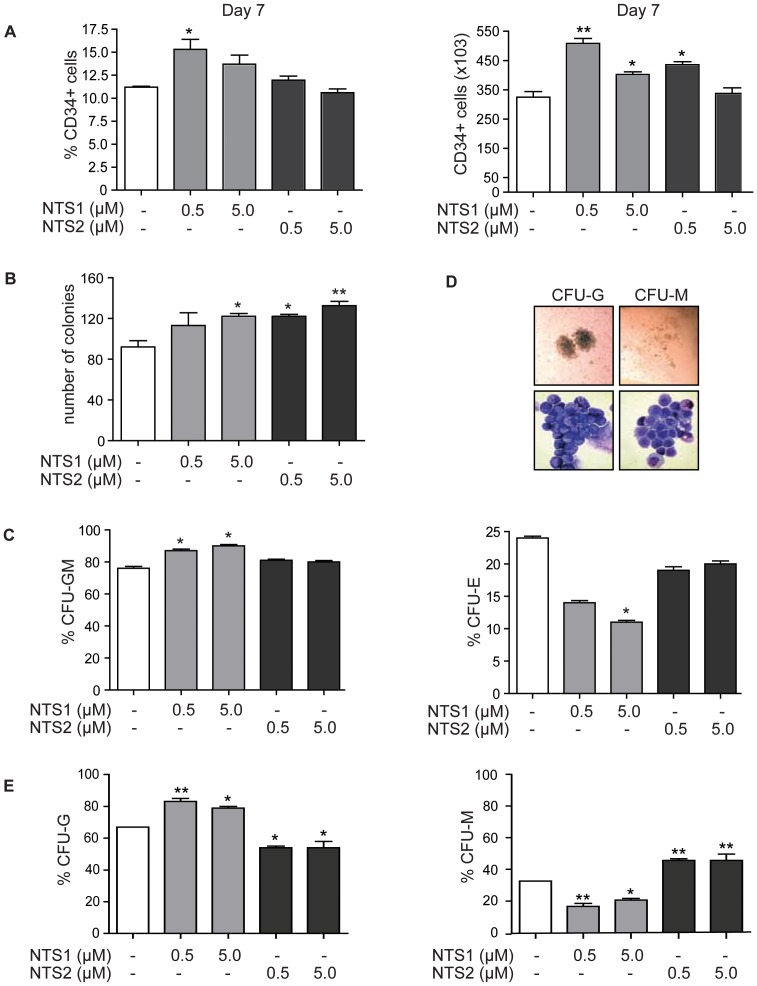
NTS1 and NTS2 differentially stimulate myeloid progenitor expansion and differentiation. CD34+ cells were cultured in the presence of G-CSF to induce neutrophil differentiation. Cells were cultured in the absence or presence of 0.5 µM or 5 µM NTS1 or NTS2. At day 7, FACS using a PE-labelled human-CD34 antibody measured CD34+ expression. For CFU assays, CD34+ cells were cultured in the presence of G-CSF and EPO, or IL6 to induce myeloid colony formation during 11 days. Data were expressed as the percentage and absolute number of CD34+ positive cells (**A**) (N = 3), the number of colonies after exposure to NTS1 or NTS2 for 3 days (**B**) (N = 3), the percentage of CFU-GM and CFU-E in the absence or presence of NTS1 or NTS2 (**C**) (N = 3), or the percentage of CFU-G and CFU-M in the absence or presence of NTS1 or NTS2 (**D–E**) (N = 2). Error bars represent SEM (between experiments). *p = <0.05, **p = <0.01.

In order to characterize the effects of NTS1 and NTS2 treatment on CD34+ myeloid progenitors specifically, CD34+ cells were differentiated towards neutrophils in the absence or presence of NTS1 or NTS2. At day 3 and 7 of differentiation, the percentage and absolute number of CD34+ progenitor cells were analyzed by FACS. No significant effects at day 3 (data not shown) were observed, while treatment with NTS1 (0.5 µM) resulted in a significant increase in both the percentage and absolute number of CD34+ cells at day 7, suggesting that NTS1 stimulates myeloid progenitor expansion. Treatment with NTS2 (0.5 µM) also induced a significant increase in the number of CD34+ cells at day 7 (Figure 2A). To further investigate the effects of NTS treatment on the expansion and functional capacity of myeloid progenitors, CFU-assays were performed. In advance, CD34+ cells were cultured in the presence of SCF, FltL3, IL3, GM-CSF and G-CSF and treated with NTS1 or NTS2 (0.5 µM or 5.0 µM) for 3 days. After this time, cells (1000 per condition) were isolated from the suspension cultures and plated in methylcellulose in the presence of the previously mentioned cytokines, without additional treatment with NTS1 and NTS2. The total number of colonies was scored after 11 days. Treatment with 5.0 µM NTS1, 0.5 µM NTS2 and 5.0 µM NTS2 induced a significant increase in the number of colonies, suggesting that the isolated cell populations pretreated with both compounds contained an increased number of progenitors with myeloid colony forming potential (Figure 2B).

To further evaluate the effects of NTS1 and NTS2 on differentiation and lineage choice, we performed CFU-assays with specific cytokine combinations. To investigate whether NTS1 and NTS2 treatment has effect on myeloid lineage choice, EPO was added to the cytokine cocktail, followed by plating of cells at day 0 in the absence or presence of NTS1 and NTS2 and colonies were scored after 11 days. Treatment with NTS1 resulted in a significantly decreased percentage of CFU-E, accompanied by a significantly increased percentage of CFU-GM (Figure 2C), suggesting that NTS1 treatment stimulates differentiation towards the GM-lineage. In contrast, we observed no significant effects upon treatment with NTS2. To determine the effects of NTS1 and NTS2 specifically on the differentiation of GMP in granulocyte colonies (CFU-G) or monocyte/macrophage colonies (CFU-M), CD34+ cells were plated in methylcellulose in the presence of SCF, IL-3 and IL-6. The number of CFU-G and CFU-M was scored after 11 days and confirmed by cytospin analysis of isolated colonies (Figure 2D). Upon treatment with NTS1 we observed a significant increase in the percentage of CFU-G colonies, and significant decrease in the percentage of CFU-M, while NTS2 treatment resulted in a significantly increased percentage of CFU-M, and decreased percentage of CFU-G (Figure 2DE). Together these data suggest that treatment with both NTS1 and NTS2 induces expansion of myeloid progenitors and stimulates differentiation towards the GM-lineage. In addition NTS1 and NTS2 appear to have compound-specific effects on differentiation of GMP towards the granulocyte or monocyte/macrophage lineage.

### NTS1 and NTS2 treatment does not affect terminal neutrophil differentiation

**Figure 3:**
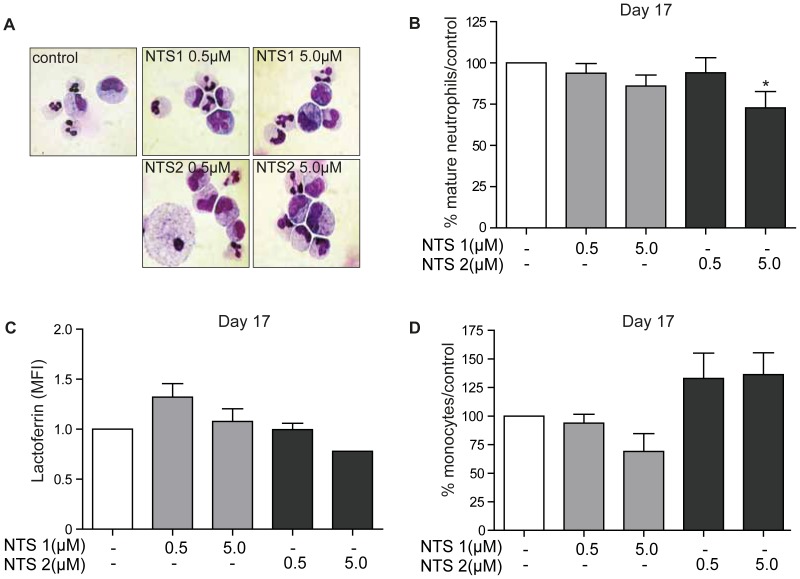
Treatment with NTS1 and NTS2 does not affect terminal neutrophil differentiation. CD34+ cells were cultured in the presence of G-CSF to induce neutrophil differentiation during 17 days. Cells were cultured either in the absence or presence of NTS1 (0.5–5 µM) or NTS2 (0.5–5 µM). After 17 days of neutrophil differentiation was determined by cytospin analysis (**A**) and FACS analysis or intracellular lactoferrin expression. Data were expressed as the percentage of mature neutrophils (banded or segmented nuclei) (N = 3) (**B**) the mean lactoferrin expression (MFI) (**C**) (N = 3), and the percentage of mature monocytes (**C**). Error bars represent SEM (between experiments). *p = <0.05, **p = <0.01.

In order to determine the effects of treatment with NTS1 and NTS2 on terminal neutrophil differentiation, CD34+ cells were differentiated towards neutrophils for 17 days in the absence or presence of NTS1 and NTS2. Neutrophil differentiation was determined based on both cytospin analysis (Figure 3A), and lactoferrin staining. Terminally differentiated neutrophils were characterized as cells containing either banded or segmented nuclei. Treatment with NTS1 and 0.5 µM NTS2 resulted in no significant effects on neutrophil differentiation, while treatment with 5.0 µM NTS2 resulted in a small, but significant decrease in the percentage of mature neutrophils (Figure 3B), which was not accompanied by decreased lactoferrin expression (Figure 3C). A moderate increase in the percentages of mature monocytes was also observed (Figure 3D). Together, these data suggest that terminal neutrophil differentiation is unaffected by NTS1 and is moderately affected by NTS2 treatment in a concentration-dependent manner. In addition, in agreement with our results from the CFU assays, these data indicate that NTS2 treatment appears to favor differentiation towards mature monocytes/macrophages.

### NTS1 and NTS2 treatment stimulates myeloid colony forming capacity in patient-derived bone marrow cells

**Figure 4:**
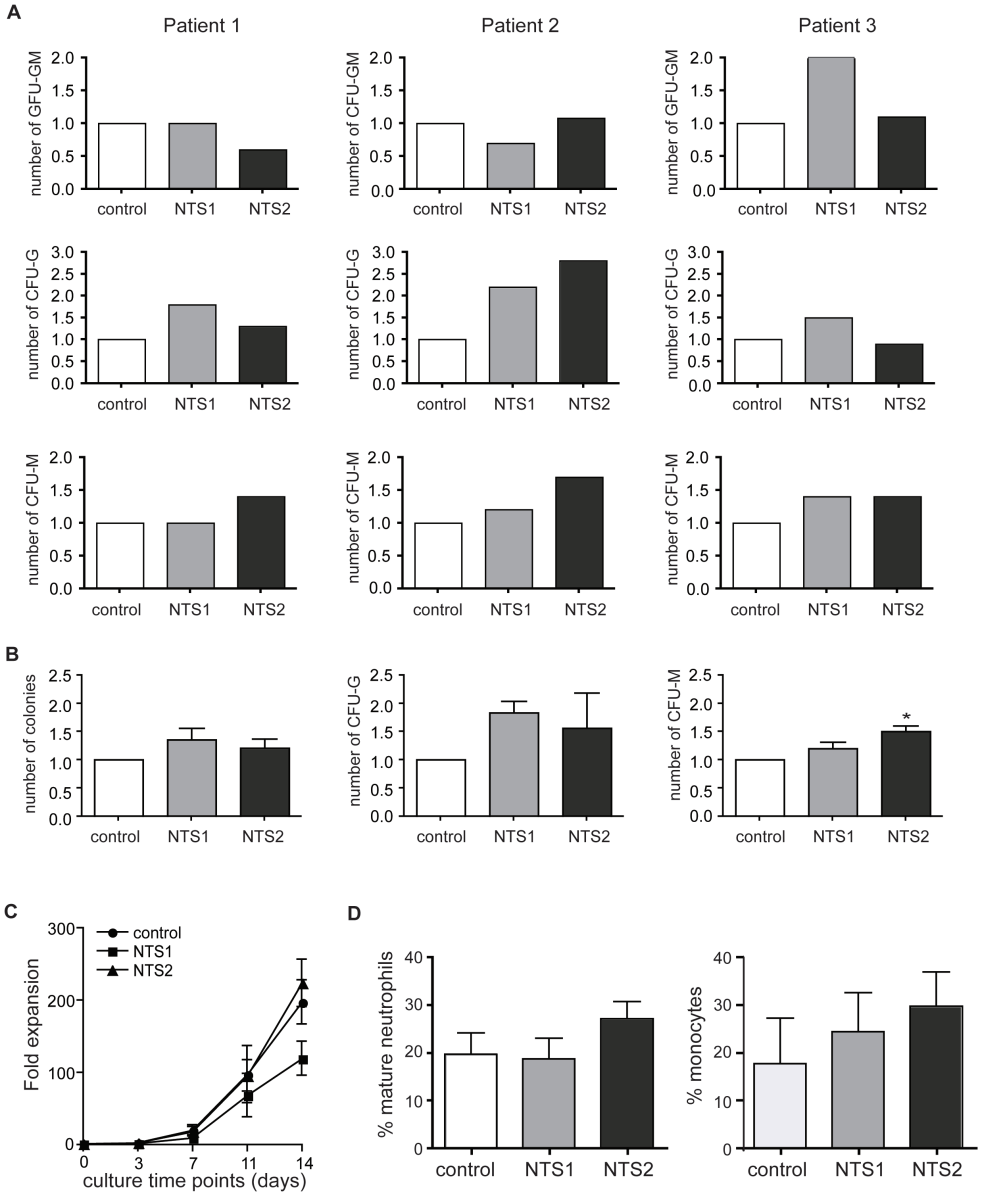
NTS treatment stimulates myeloid colony forming capacity in patient bone marrow cells. CD34+ cells were cultured in the presence of G-CSF to induce neutrophil differentiation in the absence or presence of 0.5 µM (patient 1) or 1.0 µM (patient 2–3) NTS1 and NTS2. Cells were isolated after 3 days for CFU assays in the presence of G-CSF during 11 days. Data were expressed as the number of CFU-GM (A, upper panel), CFU-G (**A, middle panel**) and CFU-M (**A, lower panel**) for each patient and for all patients together (**B**). Progenitor expansion and terminal differentiation was evaluated after 14 days, Data were expressed as fold induction (**C**) and the percentage of mature neutrophils and monocytes (**D**). Error bars represent SEM (between patients). *p = <0.05.

To investigate the effects of NTS compounds on myelopoiesis in patients suffering from myeloid disorders (one patient with RCC, two patients with myelosuppression after chemotherapy for AML), we cultured BM-derived CD34+ cells in the absence or presence of NTS1 and NTS2 (0.5–1.0 µM). Patient characteristics are summarized in Table S1. To analyze the effects on colony forming capacity and myeloid differentiation, we performed CFU assays utilizing myeloid progenitor cells isolated after three days of suspension culture. We observed differential effects on the number of CFU-GM, while NTS1 treatment increased the number of CFU-G and NTS2 treatment significantly increased the number of CFU-M (Figure 4A–B). Interestingly, treatment with NTS2 in cells from patient 2 induced a 2.8-fold increase in the number of CFU-G (Figure 4A middle lane, middle panel). While treatment with NTS1 and NTS2 in cells from patient 3 induced a moderate increase in CFU-G and CFU-M respectively, NTS1 treatment induced a 2-fold increase in the number of CFU-GM (Figure 4A, upper panel). Next, we investigated the effects of NTS1 and NTS2 treatment on neutrophil progenitor expansion and terminal neutrophil differentiation. In contrast with the effects in UCB-derived cells, we observed no significant effects on progenitor expansion (Figure 4C) upon treatment with NTS1 and NTS2. In agreement with the effects of NTS1 and NTS2 in UCB-derived cells, we observed no significant effects on the percentage of mature neutrophils and monocytes after 14 days of differentiation (Figure 4D) and no differences in intracellular lactoferrin expression (data not shown), suggesting that NTS1 and NTS2 treatment does not affect terminal neutrophil differentiation. Together, these data suggest NTS treatment stimulates the expansion of myeloid CFU in patients suffering from myeloid disorders, specifically patients with chemotherapy induced myelosuppression, while terminal neutrophil and monocyte differentiation is not affected,

### NTS1 and NTS2 treatment stimulates myelopoiesis *in vivo* and is accompanied by modulation of C/EBPα and p38MAPK activity

**Figure 5:**
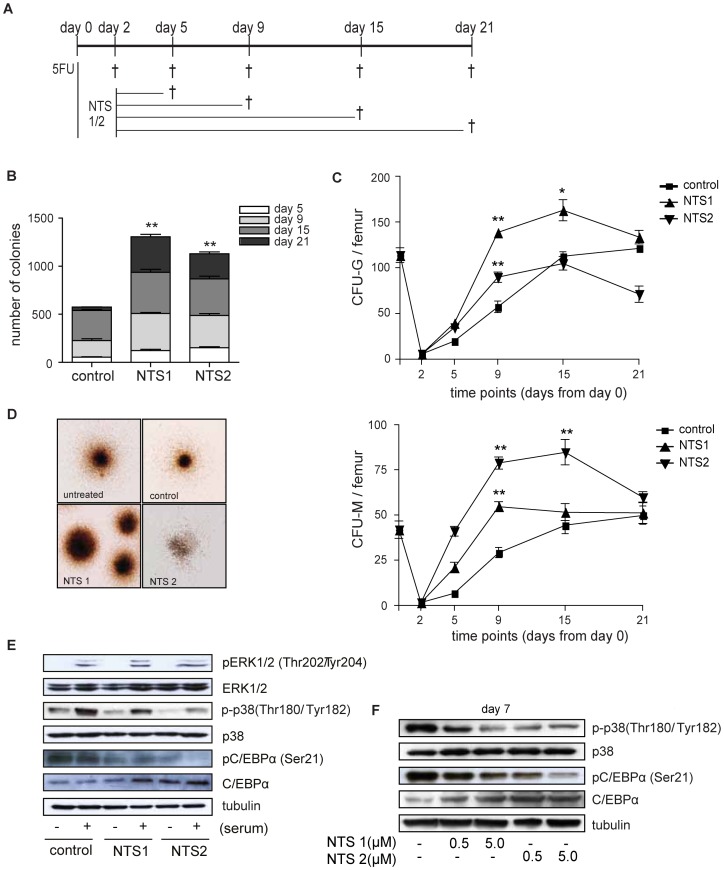
NTS1 and NTS2 treatment stimulates myelopoiesis and affects C/EBPα and p38MAPK activity. BALB/c mice were treated with 150 mg/kg 5FU at day 0, followed by treatment with 1 mg/kg NTS1 or NTS2 once a week. Mice were treated 2, 5, 9, 15, or 21 days (3 per group). Mice only treated with 5FU were used as control (2 per group) (**A**). Bone marrow mononuclear cells were cultured in the presence of rmGM-CSF and rmG-CSF to induce myeloid colony formation during 7 days. Data were expressed as the cumulative number of colonies (**B**) and the number of CFU-G and CFU-M per femur at day 2, 5, 9, 15 and 21 (**C–D**). Error bars represent SEM (between mice). *p = <0.05, **p = <0.01. Data are representative for 2 independent experiments. Ba/F3 cells were starved overnight in the presence of 0.5% FCS. Cells were left untreated or treated with NTS1 or NTS2 for 30 minutes, before stimulation with 10% FCS for 15 minutes. CD34+ cells were cultured in the presence of G-CSF for 6 days in the absence or presence of 0.5 or 5.0 µM NTS1 or NTS2. Protein lysates were prepared and Western Blot analysis was performed with an antibody against phosphorylated ERK1/2, phosphorylated p38MAPK and phosphorylated C/EBPα. Antibodies against ERK1/2, p38MAPK, C/EBPα and tubulin were used as controls (**E–F**). Data are representative for 3 independent experiments.

To investigate whether treatment with NTS1 and NTS2 also stimulates myeloid development *in vivo*, 6–8 weeks old BALB/c mice were treated with 5FU to establish complete bone marrow depletion, followed by treatment with NTS1 or NTS2 (1 mg/kg) once a week. Mice (three per group) were treated 2, 5, 9, 15 or 21 days, after which they were sacrificed. Bone marrow mononuclear cells were isolated and CFU assays were performed to analyze colony forming capacity and myeloid differentiation (Figure 5A). Colonies were scored after 7 days. We observed a significant increase in the absolute number of myeloid colonies derived from mononuclear cells from mice sacrificed at day 9 and 15 after treatment with either NTS1 or NTS2 (Figure 5B). Treatment with NTS1 resulted in a significant increase in CFU-M and dramatic increase in CFU-G suggesting that NTS1 treatment favors differentiation towards the granulocytic lineage *in vivo*. In contrast, NTS2 treatment resulted in a dramatic increase in the number of CFU-M and less pronounced increase in CFU-G, suggesting that NTS2 favors differentiation towards the monocyte/macrophage lineage *in vivo* (Figure 5C–D). Together, these data suggest that NTS1 and NTS2 treatment stimulate myeloid recovery following bone marrow depletion by 5FU. To investigate the underlying molecular mechanism for the effects of NTS treatment on myeloid development we analyzed the effects of NTS1 and NTS2 on p38 MAPK and ERK1/2 activation. We first analyzed their activation status utilizing bone marrow derived murine (Ba/F3) cells and subsequently utilizing UCB-derived neutrophil progenitors after 6 days of myeloid differentiation. Treatment with NTS1 and NTS2 resulted in a decrease in phosphorylation of p38MAPK (Thr180/Tyr182) and its substrate C/EBPα (Ser21) in BaF/3 cells and neutrophil progenitors, while we observed no effects on phosphorylation of ERK1/2 (Figure 5E–F). In addition we observed a profound increase in C/EBPα expression. Since the expression of C/EBPα is essential for granulocyte/macrophage lineage development and dephosphorylation of C/EBPα (serine 21) results in C/EBPα activation during neutrophil development [18], our data suggest that NTS1 and NTS2 stimulate myeloid differentiation through C/EBPα-dependent mechanisms. Increased C/EBPα activation can be, at least partly, explained by inhibition of p38MAPK activity.

## Discussion

In the present study, we have investigated the effects of the nitrostyrene derivatives NTS1 and NTS2 on myelopoiesis. While the predicted functional differences between NTS1 and NTS2 are small, these could involve increased protein binding of the allosteric site by NTS2 [11]. Our data demonstrate that treatment with NTS1 and NTS2 stimulates the expansion of myeloid progenitors accompanied by specific effects on differentiation of myeloid progenitors towards the granulocytic lineage (favored by NTS1) and the monocyte/macrophage lineage (favored by NTS2) *in vitro* and *in vivo*. These effects were accompanied by dephosphorylation of p38MAPK and C/EBPα, and increased C/EBPα expression.

Regulation of neutrophil and monocyte/macrophage cell fates largely depends on C/EBPα and PU.1 activity levels and the presence of G-CSF. Briefly, C/EBPα activity is regulated by G-CSF signaling, and an increased C/EBPα:PU.1 ratio favors neutrophil over monocyte/macrophage differentiation [19]–[21]. The effects we observed on phosphorylation and expression of C/EBPα in myeloid progenitor cells upon treatment with NTS1 and NTS2, suggest a potential underlying mechanism for stimulation of GM-lineage development by these compounds. In addition, since neutrophil differentiation *in vitro* is performed in the presence of high levels of G-CSF, this explains why we did not observe significant effects on terminal neutrophil differentiation upon treatment with NTS2.

The expression and function of transcription factors is regulated by post-translational modifications, including phosphorylation by MAPK. Phosphorylation of C/EBPα can be regulated by the acitivities of ERK1/2 and p38MAPK [18],[22]. While we observed no significant effects of NTS treatment on ERK1/2 activity, treatment with NTS1 and NTS2 resulted in dephosphorylation of C/EBPα accompanied by decreased phosphorylation of p38MAPK in myeloid progenitors, suggesting that the effects we observe can at least be partly explained by p38MAPK activity. The precise role of p38MAPK in myeloid development remains unclear. Aberrant p38MAPK activity has been demonstrated in bone marrow-derived myeloid progenitors from patients with MDS or aplastic anemia, which has been explained by increased activity of the myelosuppressive cytokines IFNγ and TNFα, which stimulate p38MAPK activity. Inhibition of p38MAPK by pharmacological inhibitors in these cells resulted in increased erythroid (BFU-E) and granulocyte/macrophage (CFU-GM) colony formation and inhibition of apoptosis of myeloid progenitor cells [22]–[25]. In agreement with our data, this suggests that inhibition of p38MAPK stimulates the expansion of myeloid progenitors.

While other working mechanisms of nitrostyrene derivatives have been suggested, these compounds are best known as serine/threonine phosphatase inhibitors (predominantly PP2A) [10],[11]. Since aberrant serine/threonine phosphatase activity plays a role in tumor development and progression [26],[27], phosphatase inhibitors, including nitrostyrene derivatives, are being investigated as a new group of anticancer drugs. Interestingly, treatment with nitrostyrenes in our study in non-malignant myeloid cells resulted in dephosphorylation of C/EBPα and p38MAPK, suggesting either inhibition of serine/threonine kinase activity, such as p38MAPK, or stimulation of serine/threonine phosphatase activity, such as wild-type p53-induced phosphatase 1 (WIP1). Intriguingly, these findings are in contrast with the results of NTS treatment in malignant cells, suggesting cell specific effects or reflecting an altered balance between kinase and phosphatase activity in normal versus malignant cells. This hypothesis is supported by previous studies, demonstrating that regulation of p38MAPK activity by PP2A results in differential effects on cell survival in tumor cells and normal immune cells [28]–[30].

In summary we have demonstrated that the nitrostyrene compounds NTS1 and NTS2 stimulate the expansion of myeloid progenitors *in vitro* and dramatically improve myeloid recovery after chemotherapy-induced bone marrow depletion *in vivo*.

NTS1 and NTS2 may regulate myeloid differentiation through activation of C/EBPα, which could at least be partly explained by of inhibition of p38MAPK activity. We observed moderate effects on myeloid colony formation and differentiation in bone marrow-derived cells from patients suffering from myeloid disorders. Increased knowledge concerning nitrostyrene compounds might contribute to the development of alternative therapeutic modalities for the treatment of bone marrow failure syndromes, hypocellular MDS/RCC or chemotherapy-induced aplasia.

## Supporting Information

Table S1 Patient characteristics. CD34+ cells were isolated from BM specimen of patients suffering from myeloid disorders, including one patient with RCC at a yearly control visit, and two AML patients with chemotherapy-induced BM suppression. *according to the DCOG (Dutch Child Oncology Group), ^§^RCC indicates refractory cytopenia of childhood, ^#^treatment according to the Dutch-Belgium Pediatric AML (DB-AML1) protocol.
(DOCX)
