# Supplementary material for: Differential Effects of Nitrostyrene Derivatives on Myelopoiesis Involve Regulation of C/EBPα and p38MAPK Activity
Source: PLoS One. 2014 Mar 10;9(3):e90586. doi: 10.1371/journal.pone.0090586 (PMC3948686; doi:10.1371/journal.pone.0090586)
Supplement: Table S1 — Patient characteristics. CD34+ cells were isolated from BM specimen of patients suffering from myeloid disorders, including one patient with RCC at a yearly control visit, and two AML patients with chemotherapy-induced BM suppression. *according to the DCOG (Dutch Child Oncology Group), §RCC indicates refractory cytopenia of childhood, #treatment according to the Dutch-Belgium Pediatric AML (DB-AML1) protocol. (DOCX) [file pone.0090586.s001.docx]

**Supplementary table 1.**
